# Extended Coagulation Profiling in Isolated Traumatic Brain Injury: A CENTER-TBI Analysis

**DOI:** 10.1007/s12028-021-01400-3

**Published:** 2021-12-16

**Authors:** Julia K. Böhm, Victoria Schaeben, Nadine Schäfer, Helge Güting, Rolf Lefering, Sophie Thorn, Herbert Schöchl, Johannes Zipperle, Oliver Grottke, Rolf Rossaint, Simon Stanworth, Nicola Curry, Marc Maegele, CENTER-TBI Participants and Investigators, Cecilia Åkerlund, Krisztina Amrein, Nada Andelic, Lasse Andreassen, Audny Anke, Anna Antoni, Gérard Audibert, Philippe Azouvi, Maria Luisa Azzolini, Ronald Bartels, Pál Barzó, Romuald Beauvais, Ronny Beer, Bo-Michael Bellander, Antonio Belli, Habib Benali, Maurizio Berardino, Luigi Beretta, Morten Blaabjerg, Peter Bragge, Alexandra Brazinova, Vibeke Brinck, Joanne Brooker, Camilla Brorsson, Andras Buki, Monika Bullinger, Manuel Cabeleira, Alessio Caccioppola, Emiliana Calappi, Maria Rosa Calvi, Peter Cameron, Guillermo Carbayo Lozano, Marco Carbonara, Simona Cavallo, Giorgio Chevallard, Arturo Chieregato, Giuseppe Citerio, Iris Ceyisakar, Hans Clusmann, Mark Coburn, Jonathan Coles, Jamie D. Cooper, Marta Correia, Amra Čović, Nicola Curry, Endre Czeiter, Marek Czosnyka, Claire Dahyot-Fizelier, Paul Dark, Helen Dawes, Véronique De Keyser, Vincent Degos, Francesco Della Corte, Hugo den Boogert, Bart Depreitere, Đula Đilvesi, Abhishek Dixit, Emma Donoghue, Jens Dreier, Guy-Loup Dulière, Ari Ercole, Patrick Esser, Erzsébet Ezer, Martin Fabricius, Valery L. Feigin, Kelly Foks, Shirin Frisvold, Alex Furmanov, Pablo Gagliardo, Damien Galanaud, Dashiell Gantner, Guoyi Gao, Pradeep George, Alexandre Ghuysen, Lelde Giga, Ben Glocker, Jagoš Golubovic, Pedro A. Gomez, Johannes Gratz, Benjamin Gravesteijn, Francesca Grossi, Russell L. Gruen, Deepak Gupta, Juanita A. Haagsma, Iain Haitsma, Raimund Helbok, Eirik Helseth, Lindsay Horton, Jilske Huijben, Peter J. Hutchinson, Bram Jacobs, Stefan Jankowski, Mike Jarrett, Ji-yao Jiang, Faye Johnson, Kelly Jones, Mladen Karan, Angelos G. Kolias, Erwin Kompanje, Daniel Kondziella, Evgenios Koraropoulos, Lars-Owe Koskinen, Noémi Kovács, Ana Kowark, Alfonso Lagares, Linda Lanyon, Steven Laureys, Fiona Lecky, Didier Ledoux, Rolf Lefering, Valerie Legrand, Aurelie Lejeune, Leon Levi, Roger Lightfoot, Hester Lingsma, Andrew I. R. Maas, Ana M. Castaño-León, Marc Maegele, Marek Majdan, Alex Manara, Geoffrey Manley, Costanza Martino, Hugues Maréchal, Julia Mattern, Catherine McMahon, Béla Melegh, David Menon, Tomas Menovsky, Ana Mikolic, Benoit Misset, Visakh Muraleedharan, Lynnette Murray, Ancuta Negru, David Nelson, Virginia Newcombe, Daan Nieboer, József Nyirádi, Otesile Olubukola, Matej Oresic, Fabrizio Ortolano, Aarno Palotie, Paul M. Parizel, Jean-François Payen, Natascha Perera, Vincent Perlbarg, Paolo Persona, Wilco Peul, Anna Piippo-Karjalainen, Matti Pirinen, Horia Ples, Suzanne Polinder, Inigo Pomposo, Jussi P. Posti, Louis Puybasset, Andreea Radoi, Arminas Ragauskas, Rahul Raj, Malinka Rambadagalla, Jonathan Rhodes, Sylvia Richardson, Sophie Richter, Samuli Ripatti, Saulius Rocka, Cecilie Roe, Olav Roise, Jonathan Rosand, Jeffrey V. Rosenfeld, Christina Rosenlund, Guy Rosenthal, Rolf Rossaint, Sandra Rossi, Daniel Rueckert, Martin Rusnák, Juan Sahuquillo, Oliver Sakowitz, Renan Sanchez-Porras, Janos Sandor, Nadine Schäfer, Silke Schmidt, Herbert Schoechl, Guus Schoonman, Rico Frederik Schou, Elisabeth Schwendenwein, Charlie Sewalt, Toril Skandsen, Peter Smielewski, Abayomi Sorinola, Emmanuel Stamatakis, Simon Stanworth, Robert Stevens, William Stewart, Ewout W. Steyerberg, Nino Stocchetti, Nina Sundström, Anneliese Synnot, Riikka Takala, Viktória Tamás, Tomas Tamosuitis, Mark Steven Taylor, Braden Te Ao, Olli Tenovuo, Alice Theadom, Matt Thomas, Dick Tibboel, Marjolein Timmers, Christos Tolias, Tony Trapani, Cristina Maria Tudora, Andreas Unterberg, Peter Vajkoczy, Shirley Vallance, Egils Valeinis, Zoltán Vámos, Mathieu van der Jagt, Gregory Van der Steen, Joukje van der Naalt, Jeroen T. J. M. van Dijck, Thomas A. van Essen, Wim Van Hecke, Caroline van Heugten, Dominique Van Praag, Thijs Vande Vyvere, Roel P. J. van Wijk, Alessia Vargiolu, Emmanuel Vega, Kimberley Velt, Jan Verheyden, Paul M. Vespa, Anne Vik, Rimantas Vilcinis, Victor Volovici, Nicole von Steinbüchel, Daphne Voormolen, Petar Vulekovic, Kevin K. W. Wang, Eveline Wiegers, Guy Williams, Lindsay Wilson, Stefan Winzeck, Stefan Wolf, Zhihui Yang, Peter Ylén, Alexander Younsi, Frederick A. Zeiler, Veronika Zelinkova, Agate Ziverte, Tommaso Zoerle

**Affiliations:** 1 Institute for Research in Operative Medicine, Faculty of Health, Department of Medicine, Witten/Herdecke University, Ostmerheimer Str. 200, Building 38, 51109 Cologne, Germany; 2 Emergency and Trauma Centre, Alfred Health, 55 Commercial Road, Melbourne, VIC 3004 Australia; 3 Department of Anesthesiology and Intensive Care, AUVA Trauma Hospital, Academic Teaching Hospital of the Paracelsus Medical University, Doktor-Franz-Rehrl-Platz 5, 5010 Salzburg, Austria; 4 Ludwig Boltzmann Institute for Experimental and Clinical Traumatology, AUVA Trauma Research Center, Donaueschingenstr. 13, 1200 Vienna, Austria; 5 Department of Anesthesiology, RWTH Aachen University Hospital, Pauwelsstraße 30, Aachen, 52074 Germany; 6 NHS Blood and Transplant, John Radcliffe Hospital, Oxford University Hospital NHS Foundation Trust, Headley Way, Headington, Oxford, OX3 9DU UK; 7 Oxford Haemophilia and Thrombosis Centre, Churchill Hospital, Oxford University Hospitals NHS Foundation Trust and NIHR BRC Haematology Theme, Old Road, Headington, Oxford, OX37LE UK; 8 Department of Traumatology, Orthopedic Surgery and Sports Traumatology, Cologne-Merheim Medical Centre, Witten/Herdecke University, Campus Cologne-Merheim, Ostmerheimer Str. 200, 51109 Cologne, Germany

**Keywords:** CENTER-TBI, Coagulopathy, Fibrin monomers, Progressive intracranial hemorrhage, Thrombin generation, Traumatic brain injury

## Abstract

**Background:**

Trauma-induced coagulopathy in traumatic brain injury (TBI) remains associated with high rates of complications, unfavorable outcomes, and mortality. The underlying mechanisms are largely unknown. Embedded in the prospective multinational Collaborative European Neurotrauma Effectiveness Research in Traumatic Brain Injury (CENTER-TBI) study, coagulation profiles beyond standard conventional coagulation assays were assessed in patients with isolated TBI within the very early hours of injury.

**Methods:**

Results from blood samples (citrate/EDTA) obtained on hospital admission were matched with clinical and routine laboratory data of patients with TBI captured in the CENTER-TBI central database. To minimize confounding factors, patients with strictly isolated TBI (iTBI) (*n* = 88) were selected and stratified for coagulopathy by routine international normalized ratio (INR): (1) INR < 1.2 and (2) INR ≥ 1.2. An INR > 1.2 has been well adopted over time as a threshold to define trauma-related coagulopathy in general trauma populations. The following parameters were evaluated: quick’s value, activated partial thromboplastin time, fibrinogen, thrombin time, antithrombin, coagulation factor activity of factors V, VIII, IX, and XIII, protein C and S, plasminogen, D-dimer, fibrinolysis-regulating parameters (thrombin activatable fibrinolysis inhibitor, plasminogen activator inhibitor 1, antiplasmin), thrombin generation, and fibrin monomers.

**Results:**

Patients with iTBI with INR ≥ 1.2 (*n* = 16) had a high incidence of progressive intracranial hemorrhage associated with increased mortality and unfavorable outcome compared with patients with INR < 1.2 (*n* = 72). Activity of coagulation factors V, VIII, IX, and XIII dropped on average by 15–20% between the groups whereas protein C and S levels dropped by 20%. With an elevated INR, thrombin generation decreased, as reflected by lower peak height and endogenous thrombin potential (ETP), whereas the amount of fibrin monomers increased. Plasminogen activity significantly decreased from 89% in patients with INR < 1.2 to 76% in patients with INR ≥ 1.2. Moreover, D-dimer levels significantly increased from a mean of 943 mg/L in patients with INR < 1.2 to 1,301 mg/L in patients with INR ≥ 1.2.

**Conclusions:**

This more in-depth analysis beyond routine conventional coagulation assays suggests a counterbalanced regulation of coagulation and fibrinolysis in patients with iTBI with hemostatic abnormalities. We observed distinct patterns involving key pathways of the highly complex and dynamic coagulation system that offer windows of opportunity for further research. Whether the changes observed on factor levels may be relevant and explain the worse outcome or the more severe brain injuries by themselves remains speculative.

## Introduction

Traumatic brain injury (TBI) remains a significant medical and socioeconomic burden for patients, relatives, and health care systems [1]. Current calculations estimate approximately 69 million cases of TBI to occur each year around the globe [2]. Apart from the initial injury, posttraumatic courses, including outcomes, may be complicated by preexisting hemostatic derangements or derangements that develop with TBI [3]. Recent observational data from the prospective multicentered Collaborative European Neurotrauma Effectiveness Research in Traumatic Brain Injury (CENTER-TBI) study have confirmed the presence of laboratory coagulopathy based on conventional coagulation assays (CCAs) in approximately 20% of all patients with isolated TBI (iTBI) on hospital admission [4]. Independent risk factors may include not only the degree of impact, shock, hypothermia, and hypotension but also preinjury use of anticoagulant and/or antiplatelet therapies [4]. Patients with preinjury anticoagulant and/or antiplatelet therapy were twice as likely to have an abnormal coagulation profile as those without premedication, and this was associated with greater expansion of hemorrhagic lesions and a higher risk of delayed traumatic intracranial hemorrhage [4, 5].

Apart from the physical impact, which typically leads to disruptions of the cerebral vasculature and the blood–brain barrier, along with hemorrhage, TBI-associated factors may further alter the overall hemostatic state, leading to disruptions in the balance between hypocoagulability and hypercoagulability, thereby exacerbating the initial injury [6]. The current definitions of post-TBI coagulopathy remain heterogeneous and are often based on CCAs, such as prothrombin time with its surrogates, prothrombin ratio and international normalized ratio (INR), and activated partial thromboplastin time (aPTT). In addition, the precise mechanisms that relate to the different phenotypes of coagulopathy seen with TBI are still incompletely understood. The aim of the present study was to further characterize alterations within the coagulation system of patients with iTBI by assessing concentration changes of selected proteins indicative for hypercoagulation and hypocoagulation beyond CCAs within the early hours of injury. In this context, further interest was given to the assessment of hemorrhagic injuries, expansion, and outcome in the presence of hemostatic abnormalities after iTBI.

## Methods

### Patients and Blood Sampling

The present study was embedded into the longitudinal observational CENTER-TBI study, which had recruited patients from 60 selected centers across Europe and Israel between December 2014 and December 2017 [7]. The study was conducted in accordance with local ethical regulations and with all relevant laws of the European Union and the country of the recruiting site, including relevant laws and regulations on the use of human materials. Informed consent by the patients and/or the legal representative/next of kin was obtained according to the local legislations.

Patient inclusion criteria were clinical diagnosis of TBI, indication for computed tomography (CT) scanning, presentation to a study center within 24 h of injury, and informed consent according to local and national requirements [7]. Patients were excluded if they had any severe preexisting neurological disorder that could have confounded the outcome assessments.

Of the 60 recruiting CENTER-TBI study sites across Europe, the following centers had collected and submitted blood samples for the present analysis: (1) Antwerp University Hospital (Belgium), (2) University of Cambridge (UK), (3) Helsingin Yliopisto Finnish Institute for Molecular Medicine (Finland), (4) University Hospitals Leuven (Belgium), (5) Karolinska University Hospital Stockholm (Sweden), (6) University Hospital of Aachen (Germany), (7) Kaunas University of Technology (Lithuania), (8) Leids Universitair Medisch Centrum (Netherlands), and (9) Turku University Central Hospital (Finland). On site, EDTA and citrated blood tubes were centrifuged at 1.500 × *g* for 10 min. Platelet poor plasma samples were aliquoted and frozen at − 80 °C directly or at − 20 °C for a maximum of 48 h before being transferred to the storage temperature of − 80 °C. Samples were sent from the local recruiting sites to the central CENTER-TBI laboratory at the University of Pécs (Hungary) first and then sent on dry ice to the receiving Institute for Research in Operative Medicine (IFOM) in Cologne, Germany, for further analysis.

### Sample Selection

On receipt, the samples were linked to the clinical and laboratory data comprehensively collected into the CENTER-TBI central database (INCF Neurobot 2.0; INCF, Stockholm, Sweden). To exclude any confounding effects by external factors (e.g., extracranial injuries and/or relevant interim anticoagulant or procoagulant therapies), further sample analysis was restricted to samples received from patients with iTBI (AIS_Brain_ 2–5) only and to those with available data on coagulation captured in the CENTER-TBI central database for the first 4 h after injury. Samples received from patients with documented preexisting neurological disorders, preinjury anticoagulant and/or antiplatelet therapy, presence of extracranial injuries (AIS_Extracranial_ > 0), and missing critical data points were excluded a priori. For further analysis, the first documented blood sample collected after local hospital admission was considered together with the clinical and laboratory data captured on the same timeline. The INR on admission captured in the CENTER-TBI central database served to distinguish two groups of samples and patients: those with (1) INR < 1.2 and those with (2) INR ≥ 1.2. An INR > 1.2 has been well adopted over time as a threshold to define trauma-related coagulopathy in general trauma populations [8].

### Clinical Data

The prospectively recorded clinical and laboratory data points in the scope of the CENTER-TBI study for patient characterization and captured in the CENTER-TBI central database included demographics, injury characteristics, medical presentation on admission (Glasgow Coma Scale score, systolic blood pressure, heart rate, temperature), scheduled emergency surgical intervention, neuroworsening, parameters for tissue injury and hypoperfusion (base excess and shock index), hemodilution (platelets, hemoglobin, and hematocrit), and outcome (death and Glasgow Outcome Score Extended [GOS-E]). Presence of progressive intracranial hemorrhage (PIH) was defined by the incidence of extradural or subdural hematoma or subarachnoid hemorrhage on the initial CT scan and increase in hemorrhage size/volume on the follow-up CT scan. Follow-up data on functional outcome, including mortality and GOS-E, were obtained 6 months post injury. A GOS-E between 1 and 4 (dead, vegetative state, low-severity disability, and high-severity disability) was considered unfavorable.

### Extended Coagulation Testing

CCAs to evaluate the coagulation status at the time point of blood sampling were performed by the Institute of Transfusion Medicine (ITM) at the Cologne-Merheim Medical Center, Cologne (Germany), and included the following: Quick’s value, INR, aPTT, thrombin time, fibrinogen, and antithrombin. The assessments beyond CCAs included coagulation factor V, VIII, IX, and XIII antigens; protein C; protein S; D-dimer; and plasminogen. Citrated plasma samples were analyzed by using HemosIL assays (Werfen, Bedford, MA) on the ACL TOP CTS 700 device (Werfen).

### Enzyme-Linked Immunosorbent Assay

Soluble plasminogen activator inhibitor 1 (PAI-1) as a marker for fibrinolysis regulation was quantified via the commercial Human Serpin E1/PAI-1 DuoSet ELISA (R&D Systems, Minneapolis, MN) according to the manufacturer’s recommendations.

### Thrombin Generation and Fibrinolysis-Regulating Markers

Thrombin generation, thrombin activatable fibrinolysis inhibitor (TAFI), antiplasmin, and fibrin monomers were assessed at the Ludwig Boltzmann Institute (Vienna, Austria). By using a quantitative fluorogenic assay (STG-BleedScreen; Stago, Asnières-sur-Seine, France) on the thrombin generation analyzer (ST Genesia; Stago), the thrombin generation parameters (1) peak height and (2) endogenous thrombin potential (ETP) were determined. TAFI and the fibrinolysis regulator antiplasmin were quantified by a colorimetric assay using the STA-Stachrom TAFI and STA-Stachrom Antiplasmin kits (Stago). The level of fibrin monomers was measured with an immunoturbidimetric technology by using the STA-Liatest FM kit (Stago). For these assays, the frozen citrated plasma samples were thawed at 37 °C for 10 min in a water bath and stored at room temperature for up to 1 h.

### Statistical Analysis

For the descriptive data analysis, metric data are presented as medians with interquartile ranges and categorical data are presented as percentages. Statistical differences in patient characteristics and coagulation factors/parameters were tested by using the Mann–Whitney *U*-test and χ^2^ test, respectively. A *p* value < 0.05 was considered statistically significant. Statistical analyses were performed by using SPSS statistics version 25 for Windows (IBM Corp., Armonk, NY) and GraphPad Prism version 7.00 (GraphPad Software, La Jolla, CA).

## Results

### Study Cohort Characteristics

**Fig. 1:**
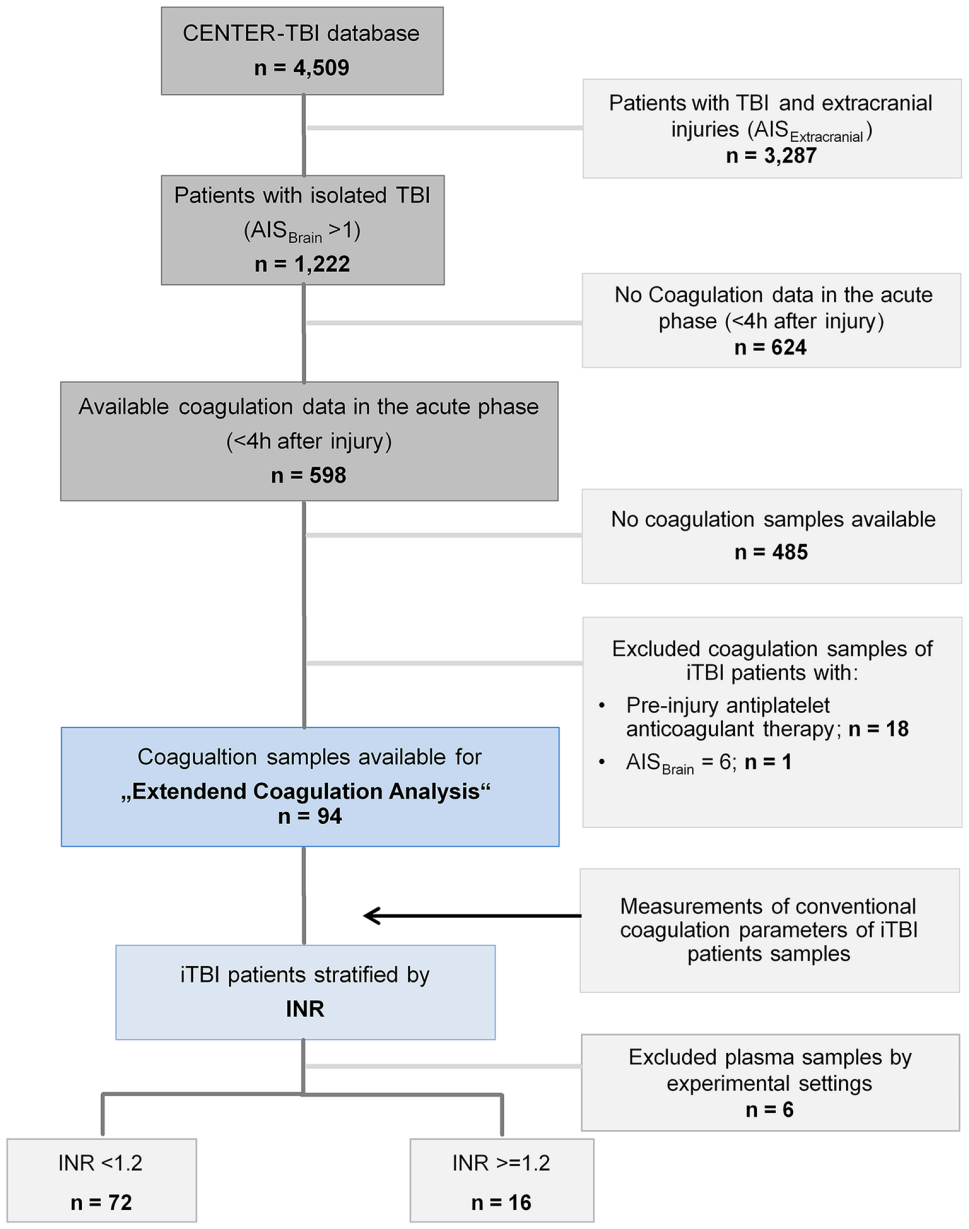
Overview of CENTER-TBI patients for selection into the present analysis to assess hemostatic abnormalities after iTBI. AIS Abbreviated Injury Scale, CENTER-TBI Collaborative European Neurotrauma Effectiveness Research in Traumatic Brain Injury, INR international normalized ratio, iTBI isolated traumatic brain injury, TBI traumatic brain injury

Overall, 4,509 patients were included in the CENTER-TBI core study database; of these, 3,287 had to be excluded for coexisting extracranial injuries and 624 for missing data (Fig. 1). Of the 598 patients with iTBI who had available coagulation data within the acute phase after TBI (< 4 h after injury), blood samples of 485 patients were missing, and to minimize interference and interfering factors, an additional 19 patients were excluded from the analysis (Fig. 1). Due to quality loss of samples during shipment and experimental settings, blood samples of another six patients had to be excluded.

The results from the blood sample analyses were matched with the clinical and routine laboratory data of corresponding patients with iTBI for a study cohort of 88 patients who were then stratified according to admission INR (Fig. 1). The basic demographics and injury characteristics of the patients included in the analysis are shown in Table 1. Almost all patients had sustained a blunt trauma mechanism, with high velocity trauma as the most common cause of injury (44%) in patients with INR ≥ 1.2, followed by ground-level falls (34%) in patients with INR < 1.2 (Table 2). In more than 90% of cases, the iTBI was moderate to severe (AIS_Brain_ ≥ 3) and closed (Table 1). Patients with INR ≥ 1.2, in general, were younger, were more shocked, and had presented with a lower Glasgow Coma Scale score. PIH as detected by CT was observed more frequent with abnormal INR. Midline shifts on CT were seen twice as frequently in patients with INR ≥ 1.2 (Table 1). Overall death increased with increasing INR as the median GOS-E at 6 months decreased (Table 1). In addition, 3.4% of the entire cohort of patients with iTBI had received hemostatic agents in the form of tranexamic acid (TXA), whereas just one patient with an INR ≥ 1.2 had been treated with TXA upon emergency department admission (Table 1).

**Table 1:** Characteristics of patients with iTBI stratified by INR (*n* = 88)

	INR <1.2 (*n* = 72)	INR ≥1.2 (*n* = 16)
Demographics		
Age, median (IQR) (years)	41.0 (30 to 57)	27.0 (22 to 54)
Age ≥ 75, n (%)	5 (6.9)	NA
Male sex, n (%)	48 (66.6)	12 (75)
Injury characteristics, n (%)		
Blunt TBI	65 (90.3)	15 (93.8)
AIS 2	6 (8.3)	1 (6.3)
AIS 3	27 (37.5)	1 (6.3)
AIS 4	21 (29.2)	6 (37.5)
AIS 5	18 (25)	8 (50)
Injuries identified on initial CT scan, n (%)		
Diffuse axonal injury	9 (12.5)	2 (12.5)
Extradural hematoma	15 (20.8)	4 (25)
Subdural hematoma	28 (38.8)	7 (43.7)
Subarachnoid hemorrhage	34 (47.2)	8 (50)
Midline shift	12 (16.7)	6 (37.5)*
Basal cistern compression	6 (8.3)	2 (12.5)
Depressed skull fracture	7 (9.7)	3 (18.8)
Severe contusion	2 (2.7)	2 (12.5)
Medical presentation at admission (ED)		
GCS, median (IQR)	14.0 (10.0 to 15.0)	12.0 (7.5 to 15.0)
SBP, median (IQR) (mm Hg)	134.0 (123 to 150)	135.5 (113 to 163)
Heart rate, median (IQR) (bpm)	84.0 (75 to 97)	82.0 (68 to 90)
Temperature, median (IQR) (°C)	36.4 (35.8 to 36.9)	36.0 (35.6 to 36.4)
Scheduled for emergency surgical intervention, n (%)	11 (15.3)	2 (12.5)
Neuroworsening, n (%)	8 (11.1)	3 (18.8)
Parameter for tissue injury and hypoperfusion, median (IQR)		
Base excess (mmol/L)	−1.1 (−2.9 to 0.55)	−2.7 (−3.7 to −0.9)
Shock index	0.61 (0.52 to 0.71)	0.64 (0.48 to 0.73)
Parameter for hemodilution, median (IQR)		
Platelet count (per nL)	220.5 (181.5 to 273.5)	211.5 (177.8 to 235.3)
Hemoglobin (g/dL)	13.9 (12.8 to 14.5)	13.4 (12.1 to 14.4)
Hematocrit	41.0 (38.0 to 44.0)	44.0 (37.7 to 45.7)*
ED therapy, n (%)		
Blood products (e.g., RBC, FFP, PC, WB)	NA	NA
Hemostatic agents		
TXA	2 (2.7)	1 (6.3)
Outcomes		
Death (overall), n (%)	7 (9.7)	3 (18.8)
GOS-E (6 months), median (IQR)	7 (6 to 8)	5 (2 to 7.5)

Neuroworsening was defined as follows: (1) a decrease in the GCS motor score of 2 or more points, (2) a new loss of pupillary reactivity or development of pupillary asymmetry ≥2 mm, or (3) deterioration in neurological or CT status sufficient to warrant immediate medical or surgical intervention. Shock index was calculated as follows: heart rate (bpm) divided by SBP (mm Hg). Patients with iTBI with AIS_Brain_ 6 were not included.

*AIS* Abbreviated Injury Scale, *aPTT* activated partial thromboplastin time, *CT* computed tomography, *ED* emergency department, *FFP* fresh frozen plasma, *GCS* Glasgow Coma Scale, *GOS-E* Glasgow Outcome Score Extended, *INR* international normalized ratio, *iTBI* isolated traumatic brain injury, *NA* not available, *PC* platelet concentrate, *PCC* prothrombin complex concentrate, *RBC* red blood cells, *SBP* systolic blood pressure, *TXA* tranexamic acid, *WB* whole blood

*P < 0.05, **P < 0.001, ***P < 0.0001: statistically significant differences between patients with iTBI with INR <1.2 and patients with iTBI with INR ≥1.2.

**Table 2:** Mechanism of injury in patients with iTBI stratified by INR (*n* = 44)

Injury Mechanisms	INR <1.2 (*n* = 35)	INR ≥1.2 (*n* = 9)
High-velocity trauma, n (%)	6 (17)	4 (44)
Blow to head (direct impact), n (%)	5 (14)	NA
Head against object (direct impact), n (%)	2 (5.7)	1 (11)
Ground-level fall, n (%)	12 (34)	2 (22)
Fall from height (>1 m), n (%)	8 (23)	1 (11)
Other closed head injury, n (%)	2 (5.7)	1 (11)

Data on mechanism of injury were missing for *n* = 44 patients with iTBI.

*INR* international normalized ratio, *iTBI* isolated traumatic brain injury, *NA* not available.

### Hemostatic Changes with Increasing INR

**Fig. 2:**
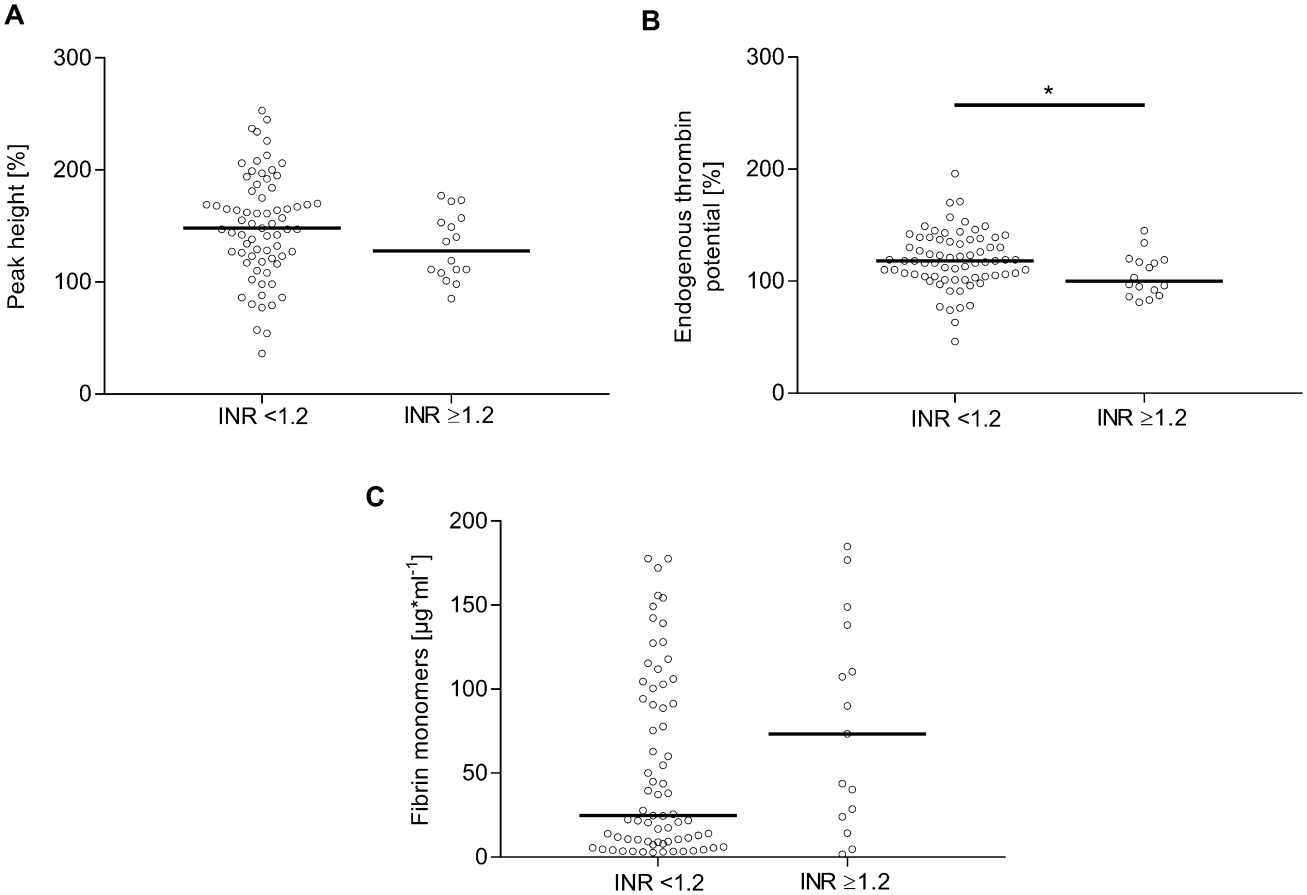
Analysis of thrombin activity and generation in plasma of patients with iTBI stratified by INR. Peak height (**A**), endogenous thrombin potential (**B**), and fibrin monomers (**C**) were measured after iTBI by using calibrated automated thrombography. Statistically significant differences between patients with iTBI with INR < 1.2 and patients with iTBI with INR ≥ 1.2 are marked with asterisks (*P < 0.05, **P < 0.001, ***P < 0.0001). INR international normalized ratio, iTBI isolated traumatic brain injury

The majority of the results from CCAs and coagulation factor concentrations, except for aPTT, thrombin time, and fibrinogen, decreased with increasing INR (Table 3). Whereas thrombin time and fibrinogen levels did not change with increasing INR, the aPTT was significantly prolonged in those patients (Table 3). The activity of coagulation factors V, VIII, IX, and XIII dropped between 10 and 25% with INR ≥ 1.2, whereas protein C and S levels fell by approximately 20%. Coagulation factor V displayed the greatest decline with 25% with INR ≥ 1.2. Also with increasing INR, thrombin generation potential became impaired, as reflected by lower peak height as well as significantly lower ETP (Fig. 2A, B). Conversely, the amount of fibrin monomers increased with increasing INR, whereas D-dimer levels rose significantly from a mean of 943 μg/L in patients with INR < 1.2 to 1,301 μg/L in patients with INR ≥ 1.2  (Fig. 2C, Table 3).

**Table 3:** Conventional coagulation parameters, coagulation factor activity and anticoagulant proteins of patients with iTBI stratified by INR (*n* = 88)

	INR <1.2 (*n* = 72)	INR ≥1.2 (*n* = 16)
Conventional coagulation parameters, median (IQR)		
Quick (%) (reference range 70–130)	101.0 (90.0–109.0)	74.0 (69.3–77.8)***
aPTT (seconds) (reference range 23–36)	28.1 (25.1–30.5)	32.0 (30.0–35.2)***
Thrombin time (seconds) (reference range 10–17)	14.0 (13.0–15.0)	14.0 (13.3–15.0)
Fibrinogen (mg/dL) (reference range 276–471)	371.0 (315.0–462.0)	389.0 (299.3–504.5)
Antithrombin (%) (reference range 83–128)	99.5 (89.3–113.0)	94.0 (84.8–103.3)*
Coagulation factors and anticoagulant proteins, median (IQR)		
Factor V (%) (reference range 62–139)	94.5 (79.3–110.8)	70.5 (51.3–78.8)***
Factor VIII (%) (reference range 50–150)	136.5 (111.8–175.5)	119.0 (100.3–169.8)
Factor IX (%) (reference range 65–150)	113.0 (99.5–128.8)	100.5 (85.3–122.3)
Factor XIII antigen (%) (reference range 75.2–154.8)	88.0 (75.0–104.0)	68.0 (61.5–83.0)**
Protein C (%) (reference range 70.0–140.0)	98.0 (86.0–112.0)	82.5 (69.3–92.0)**
Protein S (%) (reference range 63.5–149)	98.5 (81.8–118.3)	77.5 (67.2–96.3)**
D–dimer (µg/L) (reference range 0–232)	943.0 (418.0–1,300.5)	1,301.5 (966.0–6,260.5)*
Plasminogen (%) (reference range 80–133)	89.0 (79.0–97.3)	76.5 (60.0–83.8)**

Conventional coagulation parameters, coagulation factors, and anticoagulant proteins of patients with iTBI are represented as median and IQR

*aPTT* activated partial thromboplastin time, *INR* international normalized ratio, *IQR* interquartile range, i*TBI* isolated traumatic brain injury

*P < 0.05, **P < 0.001, ***P < 0.0001: statistically significant differences between patients with iTBI with INR <1.2 and those with INR ≥1.2

### Fibrinolytic Markers with Increasing INR

**Fig. 3:**
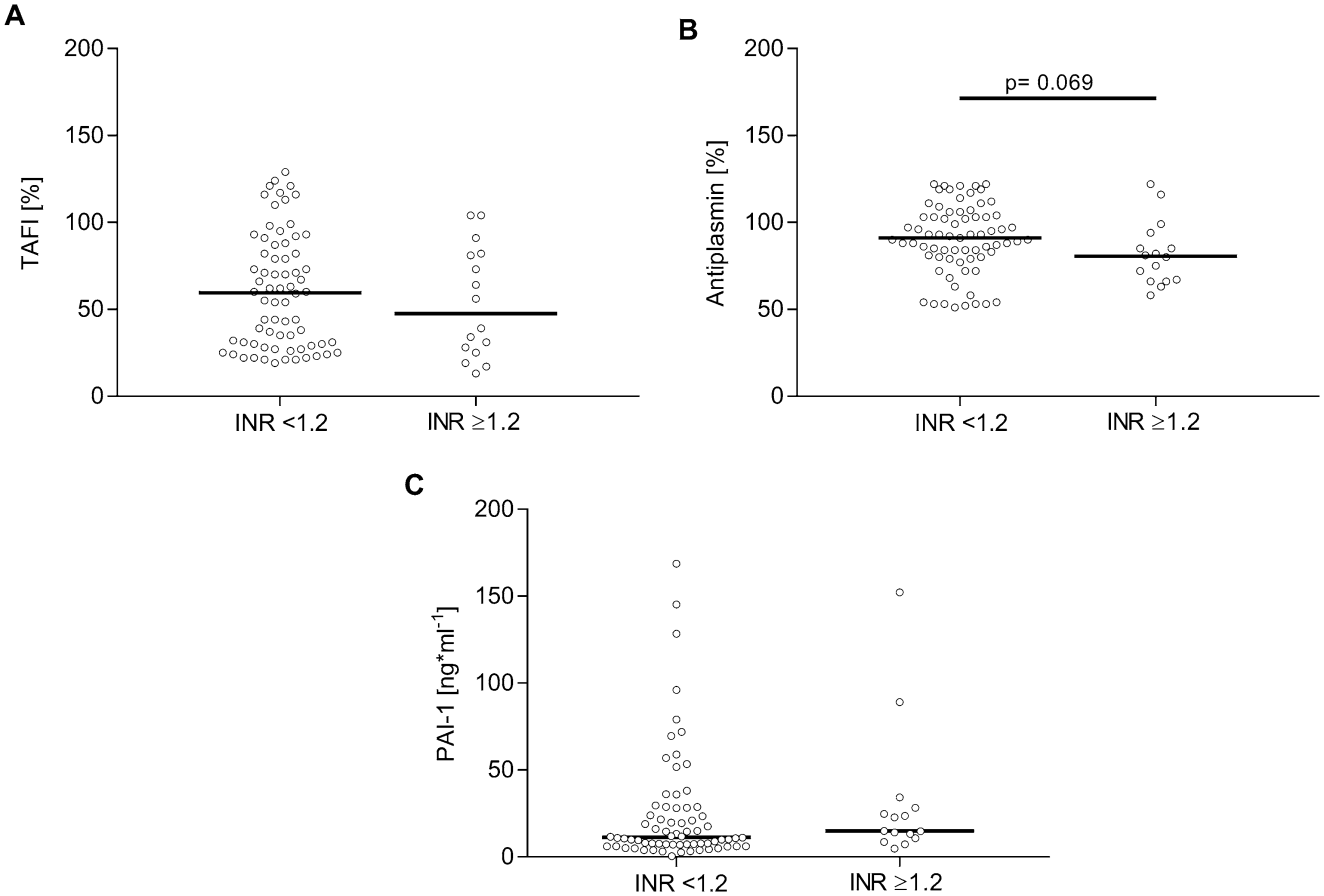
Analysis of fibrinolytic regulators in plasma of patients with iTBI stratified by INR. TAFI (**A**), antiplasmin (**B**), and PAI-1 (**C**) were measured following iTBI. Statistically significant differences between patients with iTBI with INR < 1.2 and patients with iTBI with INR ≥ 1.2 are marked with asterisks (*P < 0.05, **P < 0.001, ***P < 0.0001). INR international normalized ratio, iTBI isolated traumatic brain injury, PAI-1 plasminogen activator inhibitor 1, TAFI thrombin-activated fibrinolysis inhibitor

Fibrinolysis inhibitor PAI-1 remained largely unaltered across the two groups (Fig. 3C), whereas TAFI tended to be lower with INR ≥ 1.2  (Fig. 3A). Similarly, plasmin inhibitor antiplasmin decreased with increasing INR (Fig. 3B). In correspondence, plasminogen activity significantly decreased by 15% in patients with INR ≥ 1.2 (Table 3).

## Discussion

The results from the present study provide more detailed insights into the complex mechanisms behind the various hemostatic abnormalities occurring with and after TBI. Blood samples collected from patients with iTBI after admission to 9 of 60 recruiting CENTER-TBI study sites across Europe were submitted and used for more in-depth analyses of the coagulation system beyond those routinely used to characterize hemostatic abnormalities. The INR was used as a crude a priori way to stratify for the presence or absence of a coagulation deficit on emergency department admission [8].

Increasing INR was associated with a 1.2-fold decrease in thrombin formation peak height and ETP. This is most likely explained by the reduction of coagulation factors V and VIII in these patients because ETP is strongly dependent on these factors. The activity of coagulation factors V, VIII, and IX dropped by 10–25% of their activity with increasing INR. Coagulation factor V, in its active form, is part of the prothrombinase complex, which catalyzes the conversion of prothrombin (factor II) into thrombin (factor IIa), whereas coagulation factor IX is part of the intrinsic tenase complex, which activates prothrombinase. Also observed in the present study was a decrease in activity of coagulation factor XIII by 15% with increasing INR, which can most likely be linked to an increased activation and consumption of coagulation factors, thus coagulation factor XIII was depleted. Coagulation factor XIII is activated by thrombin to cross-link fibrin. A deficiency of coagulation factor XIII is known to worsen clot stability and increase bleeding tendency [9].

The reduced activity of thrombin-dependent factors, such as V, VIII, and XIII, may suggest impaired clot formation in iTBI with a bleeding phenotype. The authors of several studies have reported elevated thrombin generation and thrombin-generation-associated markers within 6 and 12 h following iTBI [10–12]. However, Massaro and co-workers [13] have shown a progressive and delayed procoagulant state after 48 h of injury in iTBI using thromboelastography. Thus, the decrease in thrombin activity observed in the present study indicates a consumption of thrombin, which may be caused by severe consumption of coagulation factors. Accordingly, the concentration of fibrin monomers increased with INR up to 3.0-fold with INR ≥ 1.2, whereas the activity of antithrombin decreased. A recent prospective study demonstrated that high fibrin monomer levels on admission in severe iTBI was associated with progression of hemorrhage [14]. High concentrations of fibrin monomers were observed in patients with hemostatic abnormalities (e.g., disseminated intravascular coagulation and deep vein thrombosis), and it seems promising to further evaluate the procoagulant status in these patients [15].

In a prospective study in severely injured patients with iTBI, the authors reported a reduction of antithrombin levels, which was associated with increased clot strength due to high fibrinogen concentration in the subacute phase (between days 5 and 7) after iTBI [16]. In the present study, fibrinogen levels were not depleted with increasing INR. It was concluded that a procoagulant status may occur during different phases after severe TBI [16]. Moreover, clinical data indicated the presence of severe brain injuries (AIS_Brain_ ≥ 4) in about 80% with PIH. An earlier study in critically injured trauma patients demonstrated a higher risk of thromboembolic complications with decreased antithrombin activity in those patients [17].

The balance between coagulation and fibrinolysis determines the stability of the fibrin clot. Apart from the aforementioned properties, thrombin is also involved in stabilizing the clot by activating TAFI. Alterations in thrombin generation and activation of TAFI will therefore directly affect the stabilization of clots against lysis [18, 19]. In the present study, reduced thrombin generation was associated with decreased activation of TAFI in patients with INR ≥ 1.2, which might have had a direct impact on clot protection against lysis.

In the present study, PAI-1 levels in plasma remained largely unaltered. PAI-1 is mainly produced by the endothelium and acts as a principal inhibitor of tissue plasminogen activator (tPA). According to the clinical data, patients suffered neither from hypoperfusion nor from shock, both of which are known triggers of the protein C pathway. This pathway inhibits PAI-1, leading to less inhibition of tPA, with the overall effect to promote lysis. In the experimental setting, TBI has been shown to initiate fibrinolysis, independent of shock and hypoperfusion, by releasing tPA and urokinase plasminogen activator from contused brain tissue [20]. It has also been shown that tPA activity is increased by approximately 30% within 1–3 h post TBI and returns to baseline levels by 24 h post TBI [21]. In the present study, plasminogen, which is cleaved by tPA to generate plasmin, decreased to 76% activity with increasing INR, potentially indicating enhanced conversion into plasmin. At the same time, antiplasmin activity also declined with increasing INR. These observations, together with the decreased activation of TAFI in INR ≥ 1.2, are suggestive of increased fibrinolysis, as evidenced by increasing D-dimer levels with increasing INR. It may be precluded that TXA had an influence on fibrinolysis in patients with iTBI with increasing INR because of the restricted number of patients who actually had received the agent. Endogenous release of tPA from contused brain tissue may be a distinct trigger to promote coagulopathy in iTBI apart from hemostatic abnormalities seen in the general trauma population.

The present study also suggests an activation of the protein C pathway, independent of shock and hypoperfusion, to further promote lysis, as evidenced by reductions in pathway-associated proteins (e.g., protein S, coagulation factors V and X [indirect measurement of factor X], and protein C) itself with increasing INR. Low protein C levels have previously been reported in patients with iTBI and trauma patients with hypoperfusion and were associated with poor outcome [22, 23].

There is also retrospective evidence for patients with iTBI with high D-dimer levels in plasma being at higher risk for progressive hemorrhagic injury (PHI) and worse outcomes [24, 25]. In the present study, PHI, which included the enlargement of both extradural and subdural hematoma (25% and 43.7% in patients with INR ≥ 1.2 vs. 20.8% and 38.8% in patients with INR < 1.2, respectively) and subarachnoid hemorrhage (50% in patients with INR ≥ 1.2 vs. 47.2% in patients with INR < 1.2), could be observed in patients with iTBI with elevated INR. In addition, mortality and unfavorable 6-month outcomes (GOS-E) increased with increasing INR. Several studies have previously confirmed D-dimer to predict PHI as well as correlations between elevated D-dimer levels and unfavorable outcomes, longer in-hospital stay, and higher mortality in patients with iTBI [25–28].

Although all efforts were undertaken to mirror early changes within the coagulation system in patients with iTBI, the major limitation of the present study remains the fairly large time window of patient inclusion into the CENTER-TBI core study. This precluded a stricter timeline for blood sampling after admission. In addition, blood sampling did not follow a timeline-guided protocol specifying exact time points, thereby causing variation between study centers that contributed to this study. The median time interval between injury and first blood sampling in the present study was 13.1 h (interquartile range 6.2–20.8 h). It is well accepted that hemostatic abnormalities after TBI occur quickly after the initial impact and follow dynamic patterns involving both hypercoagulative and hypocoagulative states with potential overlap [10]. Whether these findings may be interpreted as maladaptive/pathologic or represent a physiologic response to the impact remains unknown. The observation that thrombin generation and TAFI were both reduced in patients with INR ≥ 1.2 could be interpreted as a protection against lysis. Hemostatic and/or resuscitation therapies in addition to emergency procedures during acute care might have had a theoretical impact on the present results, although the focus of the analysis was given to early acute and thus mostly undiluted blood samples. In addition, prehospital care was not documented in detail in the CENTER-TBI central database. Although the present work initially aimed to present a more detailed picture of the possible underlying mechanistic changes/exchanges in hemostasis after iTBI, including outcomes, the study remains rather descriptive. Nevertheless, we observed in the present study distinct patterns involving key pathways of the highly complex and dynamic coagulation system in patients with iTBI that offer windows of opportunity for further research.

## The CENTER-TBI participants and investigators

Cecilia Åkerlund^1^, Krisztina Amrein^2^, Nada Andelic^3^, Lasse Andreassen^4^, Audny Anke^5^, Anna Antoni^6^, Gérard Audibert^7^, Philippe Azouvi^8^, Maria Luisa Azzolini^9^, Ronald Bartels^10^, Pál Barzó^11^, Romuald Beauvais^12^, Ronny Beer^13^, Bo-Michael Bellander^14^, Antonio Belli^15^, Habib Benali^16^, Maurizio Berardino^17^, Luigi Beretta^9^, Morten Blaabjerg^18^, Peter Bragge^19^, Alexandra Brazinova^20^, Vibeke Brinck^21^, Joanne Brooker^22^, Camilla Brorsson^23^, Andras Buki^24^, Monika Bullinger^25^, Manuel Cabeleira^26^, Alessio Caccioppola^27^, Emiliana Calappi ^27^, Maria Rosa Calvi^9^, Peter Cameron^28^, Guillermo Carbayo Lozano^29^, Marco Carbonara^27^, Simona Cavallo^17^, Giorgio Chevallard^30^, Arturo Chieregato^30^, Giuseppe Citerio^31, 32^, Iris Ceyisakar^33^, Hans Clusmann^34^, Mark Coburn^35^, Jonathan Coles^36^, Jamie D. Cooper^37^, Marta Correia^38^, Amra Čović ^39^, Nicola Curry^40^, Endre Czeiter^24^, Marek Czosnyka^26^, Claire Dahyot-Fizelier^41^, Paul Dark^42^, Helen Dawes^43^, Véronique De Keyser^44^, Vincent Degos^16^, Francesco Della Corte^45^, Hugo den Boogert^10^, Bart Depreitere^46^, Đula Đilvesi ^47^, Abhishek Dixit^48^, Emma Donoghue^22^, Jens Dreier^49^, Guy-Loup Dulière^50^, Ari Ercole^48^, Patrick Esser^43^, Erzsébet Ezer^51^, Martin Fabricius^52^, Valery L. Feigin^53^, Kelly Foks^54^, Shirin Frisvold^55^, Alex Furmanov^56^, Pablo Gagliardo^57^, Damien Galanaud^16^, Dashiell Gantner^28^, Guoyi Gao^58^, Pradeep George^59^, Alexandre Ghuysen^60^, Lelde Giga^61^, Ben Glocker^62^, Jagoš Golubovic^47^, Pedro A. Gomez ^63^, Johannes Gratz^64^, Benjamin Gravesteijn^33^, Francesca Grossi^45^, Russell L. Gruen^65^, Deepak Gupta^66^, Juanita A. Haagsma^33^, Iain Haitsma^67^, Raimund Helbok^13^, Eirik Helseth^68^, Lindsay Horton ^69^, Jilske Huijben^33^, Peter J. Hutchinson^70^, Bram Jacobs^71^, Stefan Jankowski^72^, Mike Jarrett^21^, Ji-yao Jiang^58^, Faye Johnson^73^, Kelly Jones^53^, Mladen Karan^47^, Angelos G. Kolias^70^, Erwin Kompanje^74^, Daniel Kondziella^52^, Evgenios Koraropoulos^48^, Lars-Owe Koskinen^75^, Noémi Kovács^76^, Ana Kowark^35^, Alfonso Lagares^63^, Linda Lanyon^59^, Steven Laureys^77^, Fiona Lecky^78, 79^, Didier Ledoux^77^, Rolf Lefering^80^, Valerie Legrand^81^, Aurelie Lejeune^82^, Leon Levi^83^, Roger Lightfoot^84^, Hester Lingsma^33^, Andrew I.R. Maas^44^, Ana M. Castaño-León^63^, Marc Maegele^85^, Marek Majdan^20^, Alex Manara^86^, Geoffrey Manley^87^, Costanza Martino^88^, Hugues Maréchal^50^, Julia Mattern^89^, Catherine McMahon^90^, Béla Melegh^91^, David Menon^48^, Tomas Menovsky^44^, Ana Mikolic^33^, Benoit Misset^77^, Visakh Muraleedharan^59^, Lynnette Murray^28^, Ancuta Negru^92^, David Nelson^1^, Virginia Newcombe^48^, Daan Nieboer^33^, József Nyirádi^2^, Otesile Olubukola^78^, Matej Oresic^93^, Fabrizio Ortolano^27^, Aarno Palotie^94, 95, 96^, Paul M. Parizel^97^, Jean-François Payen^98^, Natascha Perera^12^, Vincent Perlbarg^16^, Paolo Persona^99^, Wilco Peul^100^, Anna Piippo-Karjalainen^101^, Matti Pirinen^94^, Horia Ples^92^, Suzanne Polinder^33^, Inigo Pomposo^29^, Jussi P. Posti ^102^, Louis Puybasset^103^, Andreea Radoi ^104^, Arminas Ragauskas^105^, Rahul Raj^101^, Malinka Rambadagalla^106^, Jonathan Rhodes^107^, Sylvia Richardson^108^, Sophie Richter^48^, Samuli Ripatti^94^, Saulius Rocka^105^, Cecilie Roe^109^, Olav Roise^110,111^, Jonathan Rosand^112^, Jeffrey V. Rosenfeld^113^, Christina Rosenlund^114^, Guy Rosenthal^56^, Rolf Rossaint^35^, Sandra Rossi^99^, Daniel Rueckert^62^, Martin Rusnák^115^, Juan Sahuquillo^104^, Oliver Sakowitz^89, 116^, Renan Sanchez-Porras^116^, Janos Sandor^117^, Nadine Schäfer^80^, Silke Schmidt^118^, Herbert Schoechl^119^, Guus Schoonman^120^, Rico Frederik Schou^121^, Elisabeth Schwendenwein^6^, Charlie Sewalt^33^, Toril Skandsen^122, 123^, Peter Smielewski^26^, Abayomi Sorinola^124^, Emmanuel Stamatakis^48^, Simon Stanworth^40^, Robert Stevens^125^, William Stewart^126^, Ewout W. Steyerberg^33, 127^, Nino Stocchetti^128^, Nina Sundström^129^, Anneliese Synnot^22, 130^, Riikka Takala^131^, Viktória Tamás^124^, Tomas Tamosuitis^132^, Mark Steven Taylor^20^, Braden Te Ao^53^, Olli Tenovuo^102^, Alice Theadom^53^, Matt Thomas^86^, Dick Tibboel^133^, Marjolein Timmers^74^, Christos Tolias^134^, Tony Trapani^28^, Cristina Maria Tudora^92^, Andreas Unterberg^89^, Peter Vajkoczy ^135^, Shirley Vallance^28^, Egils Valeinis^61^, Zoltán Vámos^51^, Mathieu van der Jagt^136^, Gregory Van der Steen^44^, Joukje van der Naalt^71^, Jeroen T.J.M. van Dijck ^100^, Thomas A. van Essen^100^, Wim Van Hecke^137^, Caroline van Heugten^138^, Dominique Van Praag^139^, Thijs Vande Vyvere^137^, Roel P. J. van Wijk^100^, Alessia Vargiolu^32^, Emmanuel Vega^82^, Kimberley Velt^33^, Jan Verheyden^137^, Paul M. Vespa^140^, Anne Vik^122, 141^, Rimantas Vilcinis^132^, Victor Volovici^67^, Nicole von Steinbüchel^39^, Daphne Voormolen^33^, Petar Vulekovic^47^, Kevin K.W. Wang^142^, Eveline Wiegers^33^, Guy Williams^48^, Lindsay Wilson^69^, Stefan Winzeck^48^, Stefan Wolf^143^, Zhihui Yang^142^, Peter Ylén^144^, Alexander Younsi^89^, Frederick A. Zeiler^48,145^, Veronika Zelinkova^20^, Agate Ziverte^61^, Tommaso Zoerle^27^.

^1^Department of Physiology and Pharmacology, Section of Perioperative Medicine and Intensive Care, Karolinska Institutet, Stockholm, Sweden

^2^János Szentágothai Research Centre, University of Pécs, Pécs, Hungary

^3^Division of Surgery and Clinical Neuroscience, Department of Physical Medicine and Rehabilitation, Oslo University Hospital and University of Oslo, Oslo, Norway

^4^Department of Neurosurgery, University Hospital Northern Norway, Tromso, Norway

^5^Department of Physical Medicine and Rehabilitation, University Hospital Northern Norway, Tromso, Norway

^6^Trauma Surgery, Medical University Vienna, Vienna, Austria

^7^Department of Anesthesiology & Intensive Care, University Hospital Nancy, Nancy, France

^8^Raymond Poincare hospital, Assistance Publique – Hopitaux de Paris, Paris, France

^9^Department of Anesthesiology & Intensive Care, S Raffaele University Hospital, Milan, Italy

^10^Department of Neurosurgery, Radboud University Medical Center, Nijmegen, The Netherlands

^11^Department of Neurosurgery, University of Szeged, Szeged, Hungary

^12^International Projects Management, ARTTIC, Munchen, Germany

^13^Department of Neurology, Neurological Intensive Care Unit, Medical University of Innsbruck, Innsbruck, Austria

^14^Department of Neurosurgery & Anesthesia & intensive care medicine, Karolinska University Hospital, Stockholm, Sweden

^15^NIHR Surgical Reconstruction and Microbiology Research Centre, Birmingham, UK

^16^Anesthesie-Réanimation, Assistance Publique – Hopitaux de Paris, Paris, France

^17^Department of Anesthesia & ICU, AOU Città della Salute e della Scienza di Torino - Orthopedic and Trauma Center, Torino, Italy

^18^Department of Neurology, Odense University Hospital, Odense, Denmark

^19^BehaviourWorks Australia, Monash Sustainability Institute, Monash University, Victoria, Australia

^20^Department of Public Health, Faculty of Health Sciences and Social Work, Trnava University, Trnava, Slovakia

^21^Quesgen Systems Inc., Burlingame, California, USA

^22^Australian & New Zealand Intensive Care Research Centre, Department of Epidemiology and Preventive Medicine, School of Public Health and Preventive Medicine, Monash University, Melbourne, Australia

^23^Department of Surgery and Perioperative Science, Umeå University, Umeå, Sweden.

^24^Department of Neurosurgery, Medical School, University of Pécs, Hungary and Neurotrauma Research Group, János Szentágothai Research Centre, University of Pécs, Hungary

^25^Department of Medical Psychology, Universitätsklinikum Hamburg-Eppendorf, Hamburg, Germany

^26^Brain Physics Lab, Division of Neurosurgery, Dept of Clinical Neurosciences, University of Cambridge, Addenbrooke’s Hospital, Cambridge, UK

^27^Neuro ICU, Fondazione IRCCS Cà Granda Ospedale Maggiore Policlinico, Milan, Italy

^28^ANZIC Research Centre, Monash University, Department of Epidemiology and Preventive Medicine, Melbourne, Victoria, Australia

^29^Department of Neurosurgery, Hospital of Cruces, Bilbao, Spain

^30^NeuroIntensive Care, Niguarda Hospital, Milan, Italy

^31^School of Medicine and Surgery, Università Milano Bicocca, Milano, Italy

^32^NeuroIntensive Care, ASST di Monza, Monza, Italy

^33^Department of Public Health, Erasmus Medical Center-University Medical Center, Rotterdam, The Netherlands

^34^Department of Neurosurgery, Medical Faculty RWTH Aachen University, Aachen, Germany.

^35^Department of Anaesthesiology, University Hospital of Aachen, Aachen, Germany

^36^Department of Anesthesia & Neurointensive Care, Cambridge University Hospital NHS Foundation Trust, Cambridge, UK.

^37^School of Public Health & PM, Monash University and The Alfred Hospital, Melbourne, Victoria, Australia

^38^Radiology/MRI department, MRC Cognition and Brain Sciences Unit, Cambridge, UK

^39^Institute of Medical Psychology and Medical Sociology, Universitätsmedizin Göttingen, Göttingen, Germany

^40^Oxford University Hospitals NHS Trust, Oxford, UK

^41^Intensive Care Unit, CHU Poitiers, Potiers, France

^42^University of Manchester NIHR Biomedical Research Centre, Critical Care Directorate, Salford Royal Hospital NHS Foundation Trust, Salford, UK

^43^Movement Science Group, Faculty of Health and Life Sciences, Oxford Brookes University, Oxford, UK

^44^Department of Neurosurgery, Antwerp University Hospital and University of Antwerp, Edegem, Belgium

^45^Department of Anesthesia & Intensive Care, Maggiore Della Carità Hospital, Novara, Italy

^46^Department of Neurosurgery, University Hospitals Leuven, Leuven, Belgium

^47^Department of Neurosurgery, Clinical centre of Vojvodina, Faculty of Medicine, University of Novi Sad, Novi Sad, Serbia

^48^Division of Anaesthesia, University of Cambridge, Addenbrooke’s Hospital, Cambridge, UK

^49^Center for Stroke Research Berlin, Charité – Universitätsmedizin Berlin, corporate member of Freie Universität Berlin, Humboldt-Universität zu Berlin, and Berlin Institute of Health, Berlin, Germany

^50^Intensive Care Unit, CHR Citadelle, Liège, Belgium

^51^Department of Anaesthesiology and Intensive Therapy, University of Pécs, Pécs, Hungary

^52^Departments of Neurology, Clinical Neurophysiology and Neuroanesthesiology, Region Hovedstaden Rigshospitalet, Copenhagen, Denmark

^53^National Institute for Stroke and Applied Neurosciences, Faculty of Health and Environmental Studies, Auckland University of Technology, Auckland, New Zealand

^54^Department of Neurology, Erasmus MC, Rotterdam, the Netherlands

^55^Department of Anesthesiology and Intensive care, University Hospital Northern Norway, Tromso, Norway

^56^Department of Neurosurgery, Hadassah-hebrew University Medical center, Jerusalem, Israel

^57^Fundación Instituto Valenciano de Neurorrehabilitación (FIVAN), Valencia, Spain

^58^Department of Neurosurgery, Shanghai Renji hospital, Shanghai Jiaotong University/school of medicine, Shanghai, China

^59^Karolinska Institutet, INCF International Neuroinformatics Coordinating Facility, Stockholm, Sweden

^60^Emergency Department, CHU, Liège, Belgium

^61^Neurosurgery clinic, Pauls Stradins Clinical University Hospital, Riga, Latvia

^62^Department of Computing, Imperial College London, London, UK

^63^Department of Neurosurgery, Hospital Universitario 12 de Octubre, Madrid, Spain

^64^Department of Anesthesia, Critical Care and Pain Medicine, Medical University of Vienna, Austria

^65^College of Health and Medicine, Australian National University, Canberra, Australia

^66^Department of Neurosurgery, Neurosciences Centre & JPN Apex trauma centre, All India Institute of Medical Sciences, New Delhi-110029, India

^67^Department of Neurosurgery, Erasmus MC, Rotterdam, the Netherlands

^68^Department of Neurosurgery, Oslo University Hospital, Oslo, Norway

^69^Division of Psychology, University of Stirling, Stirling, UK

^70^Division of Neurosurgery, Department of Clinical Neurosciences, Addenbrooke’s Hospital & University of Cambridge, Cambridge, UK.

^71^Department of Neurology, University of Groningen, University Medical Center Groningen, Groningen, Netherlands

^72^Neurointensive Care , Sheffield Teaching Hospitals NHS Foundation Trust, Sheffield, UK

^73^Salford Royal Hospital NHS Foundation Trust Acute Research Delivery Team, Salford, UK

^74^Department of Intensive Care and Department of Ethics and Philosophy of Medicine, Erasmus Medical Center, Rotterdam, The Netherlands

^75^Department of Clinical Neuroscience, Neurosurgery, Umeå University, Umeå, Sweden.

^76^Hungarian Brain Research Program - Grant No. KTIA_13_NAP-A-II/8, University of Pécs, Pécs, Hungary

^77^Cyclotron Research Center , University of Liège, Liège, Belgium

^78^Centre for Urgent and Emergency Care Research (CURE), Health Services Research Section, School of Health and Related Research (ScHARR), University of Sheffield, Sheffield, UK

^79^Emergency Department, Salford Royal Hospital, Salford UK

^80^Institute of Research in Operative Medicine (IFOM), Witten/Herdecke University, Cologne, Germany

^81^VP Global Project Management CNS, ICON, Paris, France

^82^Department of Anesthesiology-Intensive Care, Lille University Hospital, Lille, France

^83^Department of Neurosurgery, Rambam Medical Center, Haifa, Israel

^84^Department of Anesthesiology & Intensive Care, University Hospitals Southhampton NHS Trust, Southhampton, UK

^85^Cologne-Merheim Medical Center (CMMC), Department of Traumatology, Orthopedic Surgery and Sportmedicine, Witten/Herdecke University, Cologne, Germany

^86^Intensive Care Unit, Southmead Hospital, Bristol, Bristol, UK

^87^Department of Neurological Surgery, University of California, San Francisco, California, USA

^88^Department of Anesthesia & Intensive Care,M. Bufalini Hospital, Cesena, Italy

^89^Department of Neurosurgery, University Hospital Heidelberg, Heidelberg, Germany

^90^Department of Neurosurgery, The Walton centre NHS Foundation Trust, Liverpool, UK

^91^Department of Medical Genetics, University of Pécs, Pécs, Hungary

^92^Department of Neurosurgery, Emergency County Hospital Timisoara , Timisoara, Romania

^93^School of Medical Sciences, Örebro University, Örebro, Sweden.

^94^Institute for Molecular Medicine Finland, University of Helsinki, Helsinki, Finland

^95^Analytic and Translational Genetics Unit, Department of Medicine; Psychiatric & Neurodevelopmental Genetics Unit, Department of Psychiatry; Department of Neurology, Massachusetts General Hospital, Boston, MA, USA

^96^Program in Medical and Population Genetics; The Stanley Center for Psychiatric Research, The Broad Institute of MIT and Harvard, Cambridge, MA, USA

^97^Department of Radiology, University of Antwerp, Edegem, Belgium

^98^Department of Anesthesiology & Intensive Care, University Hospital of Grenoble, Grenoble, France

^99^Department of Anesthesia & Intensive Care, Azienda Ospedaliera Università di Padova, Padova, Italy

^100^Dept. of Neurosurgery, Leiden University Medical Center, Leiden, The Netherlands and Dept. of Neurosurgery, Medical Center Haaglanden, The Hague, The Netherlands

^101^Department of Neurosurgery, Helsinki University Central Hospital

^102^Division of Clinical Neurosciences, Department of Neurosurgery and Turku Brain Injury Centre, Turku University Hospital and University of Turku, Turku, Finland

^103^Department of Anesthesiology and Critical Care, Pitié -Salpêtrière Teaching Hospital, Assistance Publique, Hôpitaux de Paris and University Pierre et Marie Curie, Paris, France

^104^Neurotraumatology and Neurosurgery Research Unit (UNINN), Vall d'Hebron Research Institute, Barcelona, Spain

^105^Department of Neurosurgery, Kaunas University of technology and Vilnius University, Vilnius, Lithuania

^106^Department of Neurosurgery, Rezekne Hospital, Latvia

^107^Department of Anaesthesia, Critical Care & Pain Medicine NHS Lothian & University of Edinburg, Edinburgh, UK

^108^Director, MRC Biostatistics Unit, Cambridge Institute of Public Health, Cambridge, UK

^109^Department of Physical Medicine and Rehabilitation, Oslo University Hospital/University of Oslo, Oslo, Norway

^110^Division of Orthopedics, Oslo University Hospital, Oslo, Norway

^111^Institue of Clinical Medicine, Faculty of Medicine, University of Oslo, Oslo, Norway

^112^Broad Institute, Cambridge MA Harvard Medical School, Boston MA, Massachusetts General Hospital, Boston MA, USA

^113^National Trauma Research Institute, The Alfred Hospital, Monash University, Melbourne, Victoria, Australia

^114^Department of Neurosurgery, Odense University Hospital, Odense, Denmark

^115^International Neurotrauma Research Organisation, Vienna, Austria

^116^Klinik für Neurochirurgie, Klinikum Ludwigsburg, Ludwigsburg, Germany

^117^Division of Biostatistics and Epidemiology, Department of Preventive Medicine, University of Debrecen, Debrecen, Hungary

^118^Department Health and Prevention, University Greifswald, Greifswald, Germany

^119^Department of Anaesthesiology and Intensive Care, AUVA Trauma Hospital, Salzburg, Austria

^120^Department of Neurology, Elisabeth-TweeSteden Ziekenhuis, Tilburg, the Netherlands

^121^Department of Neuroanesthesia and Neurointensive Care, Odense University Hospital, Odense, Denmark

^122^Department of Neuromedicine and Movement Science, Norwegian University of Science and Technology, NTNU, Trondheim, Norway

^123^Department of Physical Medicine and Rehabilitation, St.Olavs Hospital, Trondheim University Hospital, Trondheim, Norway

^124^Department of Neurosurgery, University of Pécs, Pécs, Hungary

^125^Division of Neuroscience Critical Care, John Hopkins University School of Medicine, Baltimore, USA

^126^Department of Neuropathology, Queen Elizabeth University Hospital and University of Glasgow, Glasgow, UK

^127^Dept. of Department of Biomedical Data Sciences, Leiden University Medical Center, Leiden, The Netherlands

^128^Department of Pathophysiology and Transplantation, Milan University, and Neuroscience ICU, Fondazione IRCCS Cà Granda Ospedale Maggiore Policlinico, Milano, Italy

^129^Department of Radiation Sciences, Biomedical Engineering, Umeå University, Umeå, Sweden.

^130^Cochrane Consumers and Communication Review Group, Centre for Health Communication and Participation, School of Psychology and Public Health, La Trobe University, Melbourne, Australia

^131^Perioperative Services, Intensive Care Medicine and Pain Management, Turku University Hospital and University of Turku, Turku, Finland

^132^Department of Neurosurgery, Kaunas University of Health Sciences, Kaunas, Lithuania

^133^Intensive Care and Department of Pediatric Surgery, Erasmus Medical Center, Sophia Children’s Hospital, Rotterdam, The Netherlands

^134^Department of Neurosurgery, Kings college London, London, UK

^135^Neurologie, Neurochirurgie und Psychiatrie, Charité – Universitätsmedizin Berlin, Berlin, Germany

^136^Department of Intensive Care Adults, Erasmus MC– University Medical Center Rotterdam, Rotterdam, the Netherlands.

^137^icoMetrix NV, Leuven, Belgium

^138^Movement Science Group, Faculty of Health and Life Sciences, Oxford Brookes University, Oxford, UK

^139^Psychology Department, Antwerp University Hospital, Edegem, Belgium

^140^Director of Neurocritical Care, University of California, Los Angeles, USA

^141^Department of Neurosurgery, St.Olavs Hospital, Trondheim University Hospital, Trondheim, Norway

^142^Department of Emergency Medicine, University of Florida, Gainesville, Florida, USA

^143^Department of Neurosurgery, Charité – Universitätsmedizin Berlin, corporate member of Freie Universität Berlin, Humboldt-Universität zu Berlin, and Berlin Institute of Health, Berlin, Germany.

^144^VTT Technical Research Centre, Tampere, Finland.

^145^Section of Neurosurgery, Department of Surgery, Rady Faculty of Health Sciences, University of Manitoba, Winnipeg, MB, Canada
